# Emotional decision-making and its dissociable components in schizophrenia and schizoaffective disorder: A behavioural and MRI investigation

**DOI:** 10.1016/j.neuropsychologia.2008.01.022

**Published:** 2008-06

**Authors:** Preethi Premkumar, Dominic Fannon, Elizabeth Kuipers, Andrew Simmons, Sophia Frangou, Veena Kumari

**Affiliations:** a Department of Psychology, Institute of Psychiatry, King's College London, De Crespigny Park, London SE5 8AF, United Kingdom; b Section of Neurobiolgy of Psychosis, Institute of Psychiatry, King's College London, London, United Kingdom; c Centre for Neuroimaging Sciences, Institute of Psychiatry, King's College London, London, United Kingdom

**Keywords:** Reward, Impulsivity, Memory, Iowa gambling task, Grey matter

## Abstract

Cognitive decision-making is known to be deficient, but relatively less is known about emotional decision-making in schizophrenia. The Iowa gambling task (IGT) is considered a reliable probe of emotional decision-making and believed to reflect orbitofrontal cortex (OFC) function. The expectancy-valence model of IGT performance implicates three dissociable components, namely, attention to reward, memory for past, relative to recent, outcomes and impulsivity in emotional decision-making. We examined IGT performance, its three components, and their grey matter volume (GMV) correlates in 75 stable patients with schizophrenia, relative to 25 healthy individuals. Patients, relative to controls, showed impaired IGT performance and poor memory for past, relative to recent, outcomes. IGT performance correlated with GMV in the OFC in controls, but not patients. There were associations between (a) attention to reward and GMV in the frontal, temporal, parietal and striatal regions in controls, and in the temporal and thalamic regions in patients, (b) memory for past outcomes and GMV in the temporal region in controls, and the frontal and temporal regions in patients, and (c) low impulsivity and greater GMV in the frontal, temporal, posterior cingulate and occipital regions in controls, and in the frontal, temporal and posterior cingulate regions in patients. Most IGT-GMV associations were stronger in controls. It is concluded that (i) poor memory, rather than less attention to reward or impulsivity, contributes to IGT performance deficit, and (ii) the relationship of IGT performance and its components with GMVs especially in the frontal and temporal lobes is lost or attenuated in schizophrenia.

## 1 Introduction

Decision-making in schizophrenia has been studied in different ways and been found to be poor in certain domains (review, Jeste, Depp, & Palmer, 2006). One type of decision-making is gambling, as assessed by the Iowa gambling task (IGT), where choices are made under conditions of uncertainty. This type of decision-making is motivated by reward and has been regarded as a type of emotional decision-making (Pecchinenda, Dretsch, & Chapman, 2006; Turnbull, Evans, Bunce, Carzolio, & O’Connor, 2005).

The IGT was first used to study emotional decision-making among people with a lesion in the orbitofrontal cortex (OFC, Bechara, Damasio, Damasio, & Anderson, 1994). On this task, participants choose cards from advantageous and disadvantageous decks, such that choosing from the disadvantageous decks is associated with greater immediate monetary reward compared to the advantageous decks, but an overall greater monetary loss compared to the advantageous decks. Choices made are likely to involve a number of cognitive and behavioural functions as the four decks of cards differ in the size and frequency of rewards/punishments. Decks A and B yield larger immediate gains ($100) than decks C and D ($50), but also yield larger losses (ranging from $100 to $1250) than decks C and D (ranging from $25 to $250), so that participants would incur a net loss over time (on average, $25 loss per card selection) by picking cards from decks A and B and a net gain over time (on average, $25 gain per card selection) by picking cards from decks C and D. Effective task performance would draw on the ability to attend to and keep online information about reward size and frequency associated with a particular deck (Yarkoni, Braver, Gray, & Green, 2005).

The expectancy-valence model of IGT (Busemeyer & Stout, 2002; Yechiam, Busemeyer, Stout, & Bechara, 2005; Yechiam, Veinott, Busemeyer, & Stout, 2007) is a cognitive model based on reinforcement learning. By simulating behaviour using computational algorithms, the expectancy-valence model allows testable predictions to be made about performance levels at different times under different conditions (Busemeyer & Stout, 2002) and in specific clinical populations (Yechiam et al., 2007). It simulates the behaviour of a person who makes a series of choices, each based on the outcome from the previous choices, without any knowledge of the actual distribution of the payoff associated with each choice (Yechiam et al., 2007). The decision-maker integrates the gains and losses experienced on each trial into a single affective reaction called a valence. The model assumes that three components determine an individual's pattern of performance on the IGT.

*Attention to reward* assesses an individual's ability to guide his/her strategy based on gains or losses received during the task. High values of this component indicate greater attention to gains rather than losses and a reward-driven performance style.

*Memory for past, relative to recent*, *outcomes* describes the degree to which expectancies of deck consequences reflect the influence of past experiences with particular decks, or rather appear to be affected by the most recent outcome with a deck. High values of this component indicate *strong* recency effects such that the most recent trials are more influential in determining the expectancy, whereas past outcomes are discounted.

*Impulsivity* evaluates the reliability with which the decision maker applies expectancies about each deck when making the card selection. High values of this component indicate that the deck with maximum expectancy will almost certainly be chosen on each trial and therefore greater choice consistency. Low values reflect an inconsistent, random and impulsive choice behaviour.

IGT performance has been studied in schizophrenia, with several studies reporting no difference between patients and controls (Ritter, Meador-Woodruff, & Dalack, 2004; Rodriguez-Sanchez et al., 2005; Shurman, Horan, & Nuechterlein, 2005; Wilder, Weinberger, & Goldberg, 1998). One study reported poorer emotional decision-making in adolescents with schizophrenia compared to healthy controls (Kester et al., 2006), and another study (Bark, Dieckmann, Bogerts, & Northoff, 2005) reported comparable emotional decision-making between patients with paranoid schizophrenia and healthy controls, but poorer emotional decision-making among patients with catatonic schizophrenia. Cognitive function and decision-making may be influenced in people with schizophrenia by emotion (Barch, 2005), and additionally by motivation (Busemeyer & Stout, 2002). There is a need for further research into motivation and emotion and their potential influences on cognitive function and decision-making in schizophrenia.

At the brain level, IGT performance is thought to reflect optimal functioning of the OFC (Bechara et al., 1994) when processing information about reward value, such as the reward size and frequency associated with a particular deck. The neural basis for reward, delineated and studied extensively among people with an addiction, is a mesolimbic thalamo-striatal prefrontal dopamingeric system (Everitt & Robbins, 2005). Dopaminergic neuronal somata in the ventral tegmentum project directly (monosynaptically) via their efferent axons to important forebrain structures that include the nucleus accumbens (ventral striatum), areas of prefrontal cortex (especially the orbitofrontal and anterior cingulate cortices) and neocortical areas, amygdala, lateral septum, thalamus, hippocampus and red nucleus of the stria terminalis (Carlson, 2007; Everitt & Robbins, 2005). Animal studies suggest that lesions in the thalamus disrupt reward-based learning (Corbit, Muir, & Balleine, 2003; Sastre & Reilly, 2006). More specifically, lesions in the medial thalamus impair learning associated with memory for reward value (Mitchell & Dalrymple-Alford, 2005). The caudate nucleus (ventral striatum) may be involved in the initial stages of emotional decision-making when reward-related contingencies are being learned (Delgado, Miller, Inati, & Phelps, 2005). The OFC is thought to hold information about reward in working memory (Gilbert & Fiez, 2004; Hikosaka & Watanabe, 2000; Schoenbaum & Setlow, 2001) and is deactivated when a reward-related cue is followed by a delay in providing the reward (Gilbert & Fiez, 2004). Lesions in the OFC impair one's ability to learn when previous reward associations no longer apply (review, Frank & Claus, 2006). The OFC has also been implicated in impulsivity. Impulsive individuals are reported to require greater OFC activity than those low on impulsivity to achieve response inhibition (Horn, Dolan, Elliott, Deakin, & Woodruff, 2003).

In this study, we examined IGT performance and its dissociable components according to the expectancy-valence model in 75 patients with schizophrenia and compared them with a group of 25 healthy controls who, on average, were matched to the patient group on age and sex. We further aimed to elucidate and compare the grey matter correlates of emotional decision-making and its dissociable components in the two groups using voxel-based morphometry. This technique allows the examination of correlations between grey matter volume and behavioural measures on a voxel-by-voxel basis across the entire brain rather than limiting the search to certain regions of interest (Gaser & Schlaug, 2003).

We hypothesized, based on previous evidence mentioned earlier, no or minimal performance impairment on the IGT in patients relative to the controls. In the case of impaired performance in patients, we expected deficient memory for previous outcomes to be the primary contributor to this deficit given that this population is frequently reported to suffer from a memory deficit (Aleman, Hijman, de Haan, & Kahn, 1999; Heinrichs & Zakzanis, 1998). We predicted stronger positive associations between IGT learning (overall) and grey matter volume in the OFC in controls than patients given previous observations of widely reported reduced grey matter volume in the prefrontal cortex (review, Molina, Sanz, Sarramea, Benito, & Palomo, 2004; Premkumar, Kumari, Corr, Sharma, 2006; Sapara et al., 2007; Shenton, Dickey, Frumin, & McCarley, 2001) including the OFC (Kuperberg et al., 2003) in chronic patients. In addition, we hypothesized that (a) greater attention to reward would be associated with greater grey matter volume in the prefrontal cortex to a greater extent in controls than patients as this region is found to respond to attention to size and frequency of rewards/punishments in healthy individuals (Yarkoni et al., 2005) and is marked by reduced grey matter volume in chronic patients (review, Shenton et al., 2001), (b) better memory for past, relative to recent, outcomes would be associated with greater grey matter volume in the mesial temporal lobe and neighbouring regions to a greater extent in controls than patients, as this region is known to be involved in memory (Cipolotti & Bird, 2006; Graham & Gaffan, 2005), and specifically in memory related to decision-making (Gutbrod et al., 2006), and is known to have reduced volume in schizophrenia patients (Wright et al., 2000), and (c) high impulsivity would be associated with lower grey matter volume in the OFC and cingulate gyrus to a greater extent in controls than patients, as these regions are activated during response inhibition in healthy individuals (Horn et al., 2003) and are areas of abnormality in schizophrenia patients (e.g., review, Fujiwara et al., 2007; Shenton et al., 2001).

## 2 Methods

### 2.1 Participants

Eighty patients were recruited to the study, of whom 75 (55 males and 20 females) provided both behavioural and MRI data. Twenty-five healthy controls (16 males and 9 females) recruited from the general public also took part. Patients were outpatient (for at least 3 months at the time of taking part in the study) with a diagnosis of schizophrenia or schizoaffective disorder. Demographic and clinical characteristics of the two study groups are presented in Table 1.

The study procedures were approved by the ethics committee of the Institute of Psychiatry and the South London and Maudsley Foundation NHS Trust. Participants provided written informed consent. All participants were compensated for their time and travel.

### 2.2 Assessments and magnetic resonance imaging

Once written consent was provided, patients had a clinical interview. Clinical diagnoses were made by an experienced consultant psychiatrist (DF) using the Structured Clinical Interview for DSM-IV (First, Spitzer, Gibbon, & Williams, 2002), who also administered the Positive and Negative Syndrome Scale (PANSS, Kay, Fiszbein, & Opier, 1987). Controls were screened for a personal or family history of mental illness and brain injury. IQ was measured in all participants using the Wechsler Abbreviated Scale of Intelligence (two text version, Wechsler, 1999) for sample characterization purposes.

#### 2.2.1 Iowa gambling task (Bechara et al., 1994)

Participants were shown four decks of cards (A, B, C and D) on a computer screen and were asked to pick a card from any of them. They were informed that each time they picked a card they would win some money as indicated by a message on the screen. Occasionally, these monetary gains were accompanied by monetary losses. The goal of the task was to maximise the monetary gain beyond the $2000 loan that participants were given to start with. They were advised that they could switch from one deck to another at any time. As some decks were associated with more monetary loss than others, the aim was to avoid the “bad” decks and to keep to the “good” decks. The task ended when the participant made 100 selections.

Overall performance was measured by the total number of selections of advantageous (C and D) cards minus the total number of selections of disadvantageous (A and B) cards within blocks of 20 selections. Dividing card selections into 5 blocks of 20 allowed us to determine the rate of learning over the course of the task. Overall learning was measured as the difference between block 5 and block 1 in the number of advantageous minus disadvantageous card selections.

#### 2.2.2 Magnetic resonance image acquisition and pre-processing

Structural MRI brain scans were acquired in all participants using a 1.5 T GE NV/i Signa system (General Electric, Milwaukee WI, USA) at the Maudsley Hospital, London. Initially, a series of sagittal fast gradient echo scout images were acquired. A 3D inversion recovery prepared fast spoiled GRASS sequence was applied to the whole brain to obtain T1-weighted images in the axial plane with 1.5 mm contiguous sections (TR = 18 ms, TI = 450 ms, TE = 5.1 ms, flip angle = 20° with one data average and a 256 × 256 × 128 voxel matrix).

Structural images were converted into ANALYZE format (ANALYZE software, BRU, Mayo Foundation, Rochester, MN) and pre-processed using Statistical Parametric Mapping—version 2 (SPM2, Wellcome Department of Imaging Neuroscience, London; http://www.fil.ion.ucl.ac.uk/spm), running in MATLAB 6.1 (MathWorks, Natick, MA). All images were manually realigned (three translations: right, forward and up; and three rotations: pitch, roll and yaw) to the anterior commissure–posterior commissure (AC–PC) line and the inter-hemispheric fissure.

### 2.3 Data reduction and scoring

#### 2.3.1 Expectancy-valence model (Yechiam et al., 2005, 2007)

The data generated from the performance on the IGT by all participants, namely trial number, the response made in each trial, the monetary loss and gain, were imported into a text file. The expectancy-valence model, which is an algorithm of the three IGT performance-derived parameters (Yechiam et al., 2005), was run on this data file in Matlab 6.1 (MathWorks, Natick, MA) and generated three components.

*Attention to reward*: It is denoted by a utility function, *u*(*t*), that allows for different weights for gains and losses, where *t* is a given trial. It is the weighted average of gains and losses for the chosen deck in a trial. It is calculated as

u(t)=W win(t)−(1−W) loss(t)

where win(*t*) is the amount of money won on trial *t*; loss(*t*) is the amount of money lost on trial *t*; and *W* is a parameter that indicates the weight given to gains versus losses.

*Memory for past*, *relative to recent*, *outcomes*: On any trial *t* the expected utility, *E*_*j*_, for deck *j* is equal to that endowed by the previous trials *E*_*j*_(*t* − 1). Expected utility is then calculated as

$$E_{j}(t)=E_{j}(t-1)+\varphi [u(t)-E_{j}(t-1)]\delta_{j}(t)$$

where *δ*_*j*_(*t*) reflects the change in expectancy if deck *j* was selected in trial *t* and *δ*_*j*_(*t*) is a weight associated with the chosen deck. When the expectancy gets updated (*δ*_*j*_(*t*) = 1), then a change occurs in the direction of the prediction error given by [*u*(*t*) − *E*_*j*_(*t*)].

*Choice consistency*: It is assumed that the consistency, denoted by *θ*(*t*), changes as a function of experience. This is formalized by a power function for the consistency change over trials:

$$\theta (t)=\left(\frac{t}{10}\right)c$$

Reliability is represented by the choice consistency parameter, denoted as *c*. The parameter *c* controls the consistency of the choice probabilities and the expectancies. When the value of *c* is low, choices are inconsistent, random, impulsive, and independent of the expectancies.

#### 2.3.2 Deriving and applying optimized normalization parameters

The structural scans were processed using the customized whole brain and tissue probability templates. The first step entailed a segmentation of the original images in native space, registering to the customized tissue probability map and correcting for image inhomogeneity, followed by the automatic brain extraction and cleaning procedure to remove non-brain tissue. The second step involved spatial normalization of the original images to the customized whole brain template using 12-parameter linear and 7 × 8 × 7 discrete cosine transform basis function non-linear transformation (Ashburner & Friston, 1999), with parameters determined from the images derived from the first step, and resliced to 1 mm × 1 mm × 1 mm voxel size to yield more accurate subsequent tissue segmentation. The spatially normalized images were then segmented into the three tissue compartments using the customized grey matter, white matter and cerebro-spinal fluid templates. Brain extraction and cleaning procedures were re-applied to the segmented normalized grey matter images to further remove extraneous brain tissue. Since the volume of some brain regions may shrink or expand as a result of non-linear spatial normalization, the cleaned grey matter images were modulated, i.e., the voxel values of each segment were multiplied by the Jacobian determinants of the deformation matrix derived during the spatial normalization step to ‘restore’ the original volume of each grey matter segment. Finally, the grey matter and white matter segments were smoothed using 12-mm FWHM isotropic Gaussian kernel to make the data conform to the Gaussian field model, underlying the structural inferences as implemented in SPM2 to render the data more normally distributed (by the central limit theorem) and to reduce the effects of individual variation in sulcal/gyral anatomy (Ashburner & Friston, 2000).

### 2.4 Statistical analysis

#### 2.4.1 Demographic characteristics

Analyses of variance (ANOVAs) and *χ*^2^-tests were performed to examine possible group differences in age, IQ and sex.

#### 2.4.2 Group differences in IGT performance

A group difference in the chronological selection of advantageous versus disadvantageous cards (overall learning) was tested using a block (5 blocks of 20 cards)-by-group (patients vs. controls) mixed factorial analysis of variance (ANOVA). The linear group-by-block interaction was examined to determine the extent of progressive switch toward more advantageous choices in the two groups. To test for possible main and interactive effects of sex, a block-by-sex-by-group mixed factorial ANOVA was also performed. In addition, a separate ANOVA was performed to test the group difference in an alternate measure of overall learning (calculated as block 5 minus block 1). Further ANOVAs examined group differences in the IGT components derived from the expectancy-valence model.

#### 2.4.3 Clinical correlates of IGT

Pearson correlations were performed to examine the relationships between IGT performance variables and the age of onset, duration of illness and symptoms (PANSS positive, negative, general psychopathology and total scores).

To determine whether only a subgroup of schizophrenia patients show atypical IGT performance, we further tested whether patients with poor IGT performance [IGT overall learning score (calculated as block 5 minus block 1) >1 standard deviation below control group score] differed from patients within the normal range of IGT performance on the above clinical measures using ANOVAs.

#### 2.4.4 MRI analysis

The grey matter correlates of overall IGT performance in the patients and controls (separately) were examined using simple linear regressions performed within SPM2 co-varying for age and sex. Similar regressions were performed in patient and control groups separately between grey matter maps and IGT components derived from the expectancy-valence model. The resulting SPM maps were thresholded for regions correlating positively or negatively (*p* ≤ 0.005, uncorrected) with IGT learning score. Small volume corrections (SVCs; sphere-shaped with search volume of 10 mm due to the anatomical extensions of the hypothesized structures, *p* ≤ 0.05) were applied during the multiple correction procedure for the hypothesized regions of interest to avoid a Type II error.

Once we determined the grey matter correlates of IGT parameters in the patient and control groups, we extracted the values representing the percentage of total grey/white matter volume under a smoothing kernel relative to the total grey/white matter volume for each participant in both groups at the maxima voxel of all the regions showing an association with IGT performance variables, calculated the corresponding *r* values for each group, and tested for statistically significant group differences in the strength of the observed IGT-grey matter associations using Fisher *Z* transformations (Howell, 2002).

We then examined whether patients had reduced grey matter volume compared to controls in the regions that showed differential associations between the two groups. For this part of the analysis, we performed a group comparison of grey matter in SPM and examined group differences in grey matter availability between the two groups applying SVCs (sphere-shaped with search volume of 12 mm) for the voxels showing a statistically different association with IGT variables between the two groups.

Finally, we compared the IGT performance/parameter-grey matter correlation patterns of patients in the normal performance range, patients showing poor performance and healthy controls using the method described to compare the correlational patterns between patients and controls.

## 3 Results

### 3.1 Demographic characteristics

Patient and control groups were matched on age and sex distribution, but patients had lower IQ than controls (Table 1).

### 3.2 Group differences in IGT performance

#### 3.2.1 Overall learning

**Fig. 1:**
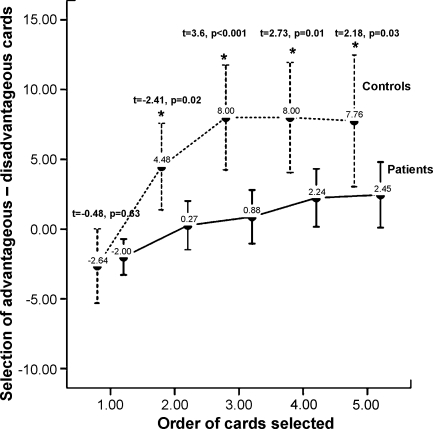
IGT block performance in patients and controls. Values represent group means, asterisks represent a significant within-block group difference (*p* < 0.01).

Patients showed reduced overall learning than controls [patients, mean (S.D.) = 4.45 (12.05); controls, mean (S.D.) = 10.40 (13.00); *F* (1,98) = 4.39, *p* = 0.04, effect size = 0.043]. The linear trend was stronger in controls [*F* (1,24) = 16.65, *p* < 0.001] than in patients [*F* (1,74) = 10.48, *p* = 0.002] as revealed by a significant linear block-by-group interaction [*F* (4,95) = 3.06, *p* = 0.02]. The quadratic trend was significant for the control group, *F* (1,24) = 17.26, *p* < 0.001, but not the patient group, *F* (1,74) = 1.64, *p* = 0.21], with a significant quadratic block-by-group interaction [*F* (1,98) = 8.17, *p* = 0.005] suggesting that the learning peak by block three observed in controls was not present in patients. Patients differed significantly from controls in choosing chards from advantageous decks from blocks two to five (see Fig. 1).

The ANOVA including sex as a between-subject factor showed no effect of sex [*F* (4,93) = 0.76, *p* = 0.55] or a block-by-group-by-sex interaction [*F* (4,93) = 1.11, *p* = 0.35].

#### 3.2.2 Parameters derived from the expectancy-valence model

Patients showed poorer memory for past, relative to recent, outcomes (as mentioned earlier higher scores indicate that past outcomes are discounted for most recent outcomes) than healthy controls during IGT performance [patients, mean (S.D.) = 0.33 (0.39); controls, mean (S.D.) = 0.17 (0.28); *F* (1,98) = 3.81, *p* = 0.05, effect size = 0.037], but no greater attention to reward [patients, mean (S.D.) = 0.45 (0.39); controls, mean (S.D.) = 0.41 (0.34); *F* (1,98) = 0.16, *p* = 0.69, effect size = 0.002] or higher impulsivity than controls [patients, mean (S.D.) = 0.32 (2.63); controls, mean (S.D.) = 0.87 (2.28); *F* (1,98) = 0.85, *p* = 0.34, effect size = 0.009].

### 3.3 IGT and clinical variables

IGT overall learning or components derived from the expectancy-valence model did not correlate with the age of onset, duration of illness and PANSS symptoms (see Table 2).

The only clinical variable to differentiate poor performing patients from patients performing in the normal range was the general psychopathology scale. Poor performing patients had higher scores [*n* = 22; mean (S.D.) = 35.41 (8.27)] than patients performing in the normal range [*n* = 53; mean (S.D.) = 31.89 (5.63)] on the PANSS general psychopathology subscale [*F* (1,74) = 4.57, *p* = 0.04]. Specifically, poor performing patients had more severe somatic concerns [mean (S.D.) = 2.68 (1.39)] than patients performing in the normal range [mean (S.D.) = 1.64 (0.88), *t* (equal variances assumed) = 3.2, d.f. = 28.2, *p* = 0.003]. Poor performing patients also had greater active social avoidance [mean (S.D.) = 3.18 (0.8)] than patients performing in the normal range [mean (S.D.) = 2.68 (1.05), *t* = 2.01, d.f. = 73, *p* = 0.05].

### 3.4 IGT correlations with grey matter volume

#### 3.4.1 Overall performance

##### 3.4.1.1 Controls

Better IGT overall learning was associated with greater grey matter volume in the left OFC (see Table 3).

##### 3.4.1.2 Patients

No hypothesized region showed a significant association with IGT overall learning.

#### 3.4.2 Components derived from the expectancy-valence model

##### 3.4.2.1 Controls

*Attention to reward*: Greater attention to reward was associated with greater grey matter volume in the right superior frontal gyrus, right superior temporal gyrus, right inferior parietal lobule and the left globus pallidus.

*Memory for past* (*relative to recent*) *outcomes*: Poor memory for past outcomes (higher values) was associated with lower grey matter volume in the right post-central gyrus and the left superior temporal gyrus.

*Impulsivity* (*choice consistency*): Low impulsivity (high choice consistency) was associated with greater grey matter volumes in the right middle frontal gyrus, left post-central gyrus, left posterior cingulate gyrus, right superior temporal gyrus, right middle temporal gyrus, right occipital gyrus, left cuneus and the right hippocampus.

##### 3.4.2.2 Patients

*Attention to reward*: Greater attention to reward was associated with greater grey matter volume in the left superior temporal gyrus and the left thalamus.

*Memory for past* (*relative to recent*) *outcomes*: Poor memory for past outcomes was associated with lower grey matter volume in the right superior frontal gyrus and the right inferior temporal gyrus.

**Fig. 2:**
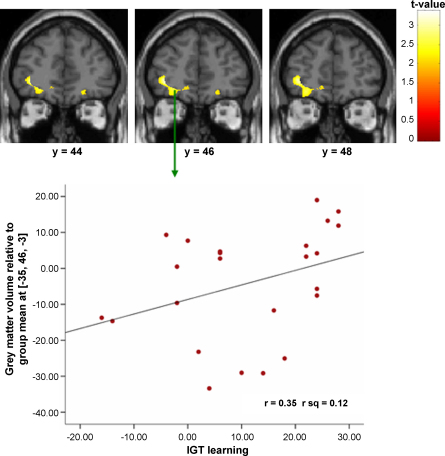
Correlation images and regression plot of a positive relationship between IGT overall learning and left orbitofrontal cortex in controls.

*Impulsivity* (*choice consistency*): Low impulsivity was associated with greater grey matter volumes in the right dorsolateral prefrontal cortex, left superior temporal gyrus and the right posterior cingulate gyri (Table 3 and Fig. 2).

#### 3.4.3 Comparison of grey matter correlates between patient and control groups

Overall IGT learning-OFC grey matter association was significantly stronger in controls than patients.

The positive association between attention to reward and grey matter volumes in the superior frontal gyrus, superior temporal gyrus and right inferior parietal lobule was stronger in controls and the association between attention to reward and left thalamus grey matter volume association was stronger in patients (see Fig. 2).

The positive association between memory for past (relative to recent) outcomes and grey matter in the right post-central gyrus was stronger in controls. The association between this performance variable and grey matter in the right inferior temporal gyrus was stronger in patients.

The negative association between impulsivity (better choice consistency) and grey matter in the right middle frontal gyrus, left post-central gyrus, right superior and right middle temporal gyri, left posterior cingulate gyrus, right occipital gyrus and left cuneus was stronger in controls (see Fig. 2).

Of all the regions associated with IGT performance or its components, patients had reduced grey matter volumes in the right superior frontal (*x* = 2, *y* = 29, *z* = 55) and right superior temporal gyri (*x* = 49, *y* = 8, *z* = −1); both regions were associated with attention to reward in controls.

#### 3.4.4 Comparison of grey matter correlates between patients in the normal IGT performance range and patients with poor IGT performance and healthy controls

The association between low impulsivity and greater grey matter volume in the right posterior cingulate gyrus (*x* = 14, *y* = −41, *z* = 40) was stronger in patients in the normal IGT performance range (*r* = 0.33, *p* = 0.03) than in the patients with poor IGT performance (*r* = −0.17, *p* = 0.53) (Fisher's *Z* = 1.62, *p* = 0.05). The two groups did not differ in any other IGT correlations with grey matter volume.

The differences in correlational patterns between patients in the normal IGT performance range and healthy controls were largely the same as those between the total patient sample and healthy controls (Table 3), except for the association between more attention to reward and greater grey matter volume in the superior temporal gyrus. This association failed to differ significantly between the patients in the normal IGT performance range and healthy controls (Fisher's *Z* = 1.23, *p* = 0.11).

## 4 Discussion

The aims of the study were (i) to investigate the specific nature of performance deficit on the IGT, and (ii) to determine whether the regions normally involved in emotional decision-making also supported emotional decision-making in people with schizophrenia.

### 4.1 Behavioural findings

Our findings revealed mild impairment in overall IGT performance in patients relative to controls. Controls switched to the advantageous decks by the second block, while patients failed to switch to advantageous decks. The effect size of this deficit was small (0.04). Shurman et al. (2005) also observed that controls (*n* = 10) selected advantageous decks by the second block, while patients (*n* = 39) were slower to switch strategy. Ritter et al. (2004) did not observe differences in IGT learning between patients and healthy controls. Participants in their study were older (mean age 47 years) and male. Difficulty with emotional decision-making may be more subtle in older male schizophrenia patients. Patients also had lower IQ than the healthy controls that may have moderated the level of impairment patients showed in emotional decision-making, as low intelligence is associated with impaired function on most cognitive domains (Heinrichs & Zakzanis, 1998; Thurston-Snoha & Lewine, 2007).

The findings derived from the expectancy-valence model showed that poor memory for past, relative to recent, outcomes, but not attention to reward or impulsivity, differentiated patients from controls. The three components relate to different features of card selection. In monitoring reward size and frequency for a given deck, a poor ability to retain information about earlier card choices may guide task performance. Motivation for the amount of money won on a trial may also influence task performance. The impulsivity (choice consistency) component focuses on the reliability of choosing a particular deck across all trials. Schizophrenia patients are known to have marked memory deficit (Aleman et al., 1999; Heinrichs & Zakzanis, 1998) that may influence good emotional decision-making.

Our patients seemed to have normal attention to reward. Most patients were receiving atypical antipsychotics that are known to have less or no adverse effects on dopamine transmission (Diaz-Mataix et al., 2005) that is required for and may preserve attention, unlike typical antipsychotics that block dopamine systems (Sawa & Snyder, 2003). Patients were also no more impulsive in their emotional decision-making than normal. Patients with poor IGT performance had more severe general psychopathology than patients within the normal range of IGT performance, more specifically with regard to active social avoidance and somatic concerns. We infer that poor emotional decision-making may influence one's beliefs about their social and physical adjustment (Hyojin, Daeyeol, Shin, & Jeanyung, 2007).

### 4.2 MRI–IGT relationships

We found the expected association between OFC grey matter volume and IGT overall learning in controls, but not in patients. The finding supports the view that OFC availability is important for normal emotional decision-making (Bechara et al., 1994). Our findings show that this relationship is lost in people with chronic schizophrenia most likely due to reduced grey matter volume in the PFC. Even the subgroup of patients with normal IGT performance did not show normal IGT-grey matter associations which would suggest that normal IGT performance is subserved by neural systems that are distinct from healthy individuals.

We also observed relationships between the functional subsystems of emotional decision-making and grey matter volume in several regions. Greater attention to reward was associated with greater grey matter volume in the right superior frontal gyrus, right superior temporal gyrus, right inferior parietal lobule and left globus pallidus in controls and with greater grey matter volume in the left thalamus and left superior temporal gyrus in patients. The association of greater attention to reward with greater grey matter volume was stronger in the left thalamus, but weaker in the right superior temporal gyrus, in addition to the right superior frontal gyrus and right inferior parietal lobule in patients relative to controls. The thalamus is involved in learning associated with memory for reward value (Frank & Claus, 2006; Mitchell & Dalrymple-Alford, 2005). We infer from our findings that the thalamus is associated with motivational and memory aspects of processing information about reward value in people with schizophrenia. Our hypothesis that greater attention to reward would be associated with greater grey matter volume in the PFC in controls was supported. It has already been observed in healthy individuals that greater event-related activity in the right superior frontal gyrus is associated with a greater number of good deck choices, where good decks had lower immediate, but greater long-term reward than bad decks, and a good ability to detect change in reward size (in both easy and difficult conditions) (Yarkoni et al., 2005). Reduced grey matter volume in the right superior frontal and superior temporal gyri in patients would suggest that these regions contribute to a lesser extent when attending to reward in patients than in healthy individuals.

Poor memory for past, relative to recent, outcomes was associated with lower grey matter volume in the right post-central gyrus and left superior temporal gyrus in controls and with lower grey matter volume in the right inferior temporal gyrus and right superior frontal gyrus in patients. The association of poor memory for past, relative to recent, outcomes with lower grey matter volume was stronger in the right inferior temporal gyrus, but weaker in the right post-central gyrus in patients relative to controls, though a significant grey matter reduction in patients was not present for these regions. The associations between poor memory for past, relative to recent, outcomes and lower left superior temporal gyrus in controls (though not different from patients) and right inferior temporal gyrus in patients suggests the involvement of the temporal lobe in memory related to emotional decision-making in both groups.

Low impulsivity (greater choice consistency) was associated with greater grey matter volumes in the right and left middle frontal gyri, right superior and middle temporal gyri, right hippocampus, left post-central gyrus, left posterior cingulate gyrus, right occipital gyrus and left cuneus in controls and with greater grey matter in the right dorsolateral prefrontal cortex, left superior temporal gyrus and right posterior cingulate gyrus in patients. The association of low impulsivity with greater grey matter volume in the right middle frontal gyrus, left post-central gyrus, left posterior cingulate gyrus, right superior and right middle temporal gyri, right occipital gyrus and left cuneus was weaker in patients relative to controls. In addition, the association of low impulsivity with greater grey matter in the right posterior cingulate gyrus was stronger in patients in the normal range of IGT performance, relative to patients with poor IGT performance. Our hypothesis that impulsivity would be associated with lower cingulate gyrus grey matter volume in controls was supported. Impulsivity is known to be related to cingulate gyrus function in healthy individuals (Brown, Manuck, Flory, & Hariri, 2006). Increased activation to response inhibition (Go/No Go task) in healthy individuals (Horn et al., 2003) has been observed in some of these regions, namely the superior temporal gyrus, cingulate gyrus and cuneus. Our findings suggest that the normal association between impulsivity and cingulate gyrus is also present in schizophrenia patients with normal IGT performance.

The associations between grey matter availability in most neural regions normally associated with impulsivity in emotional decision-making may be absent in patients with chronic schizophrenia, even among patients with a normal emotional decision-making ability. Furthermore, a region-specific grey matter deficit in the frontal and temporal lobes in schizophrenia patients may lead to the compensatory use of other regions, such as the thalamus and inferior temporal gyrus, in emotional decision-making.

### 4.3 Limitations

First, we were not able to examine the possibility of differential IGT performance in patients receiving atypical and typical antipsychotics (Beninger et al., 2003); most patients reflecting the clinical practice in our study area were treated with atypical antipsychotics and receiving typical antipsychotics is found to be associated with reduced activation of core brain reward systems, relative to atypical antipsychotics due to blockade of striatal D2 receptors. Second, we did not use tasks that specifically tested for sensitivity to reward, working memory and reversal learning, or impulsivity. Further studies using functional MRI during tasks that allow sensitivity to reward, working memory and reversal learning, or impulsivity to be tested more directly in healthy individuals as well as patients with schizophrenia are needed to extend and refine our observations.

### 4.4 Conclusions

In conclusion, poor emotional decision-making in stable outpatients with schizophrenia is likely to be due to poor memory, rather than motivational aspects of information processing. The normal IGT performance-OFC grey matter availability relationship is lost in patients with schizophrenia.

## Figures and Tables

**Table 1:** Participant characteristics

	Patient (*n* = 75)	Control (*n* = 25)	*F* (d.f.)	*p*-Value
Age, years, mean (S.D.)	37.9 (9.5)	35.4 (11.9)	1.15 (1,98)	0.29
IQ	102 (21)	119.3 (14.9)	14.51 (1,98)	<0.001
Sex, male/female (*n*)	55/20	16/9	0.79^a^	0.37

Patient characteristics				
Diagnosis (*n*)				
Paranoid	60			
Residual	4			
Catatonic	1			
Schizophrenia undifferentiated	2			
Schizoaffective disorder	8			
Age of onset, years, mean (S.D.)	23.65 (7.22)			
Duration of illness, years, mean (S.D.)	14.21 (10.16)			

PANSS, mean (S.D.)				
Positive	16.7 (4.8)			
Negative	18.1 (4.8)			
General psychopathology	32.9 (6.7)			

Total	67.6 (13.6)			

Current antipsychotic medication type				
Atypical	56			
Typical	12			
Both	6			
Non-compliant	1			
Antipsychotic medication dosage (chlorpromazine equivalents)	476.1 (324.7)			

a *χ*^2^ statistic.

**Table 2:** Correlations (*p*-value in parentheses) between IGT performance and clinical variables

	IGT overall learning	Attention to reward	Memory for recent, relative to past, outcomes	Impulsivity
Age of onset	0.09 (0.47)	−0.04 (0.77)	0.07 (0.55)	0.01 (0.95)
Duration of illness	−0.03 (0.78)	<0.001 (1.0)	−0.16 (0.18)	0.11 (0.37)
PANSS positive	0.06 (0.57)	−0.16 (0.18)	0.10 (0.39)	−0.13 (0.27)
PANSS negative	−0.11 (0.37)	0.04 (0.74)	−0.08 (0.52)	0.04 (0.72)
PANSS general psychopathology	−0.04 (0.75)	−0.14 (0.24)	−0.08 (0.47)	−0.02 (0.87)
PANSS total	−0.03 (0.78)	−0.11 (0.35)	−0.03 (0.78)	−0.04 (0.73)

PANSS: Positive and Negative Syndrome Scale.

**Table 3:** Grey matter correlates of IGT components in schizophrenia patients and controls and group comparison of the grey matter correlates

Brain region	Left/right	Brodmann area	MNI coordinates^a^			*t*-Value	Number of contiguous voxels	Corrected *p*-value	*r* (p)		Fisher's *Z* (p)
			*x*	*y*	*z*				Patient	Control	
Significant voxels in controls											
(a) Overall learning											
Orbitofrontal cortex	L		−35	46	−3	3.14	52	0.05	−0.12 (0.36)	0.35 (0.1)	2 (0.05)

(b) More attention to reward and greater grey matter volume											
Superior frontal gyrus	R	BA8	2	29	55	3.78	328	0.02	0.10 (0.46)	0.48 (0.02)	1.66 (0.05)
Superior temporal gyrus	R	BA22	49	8	−1	4.33	1172	0.006	−0.03 (0.83)	0.41 (0.04)	1.83 (0.03)
Inferior parietal lobule	R	BA7	34	−61	43	3.44	147	0.03	−0.18 (0.18)	0.61 (0.002)	3.49 (<0.001)
Globus pallidus	L		−12	5	3	3.24	270	0.04	0.15 (0.25)	0.4 (0.05)	1.07 (0.14)

(b) Poor memory for past (relative to recent) outcomes and lower grey matter volume											
Post-central gyrus	R	BA1	16	−30	76	4.07	211	0.01	0.27 (0.04)	−0.49 (0.02)	3.19 (<0.001)
Superior temporal gyrus	L	BA22	−59	−12	4	3.12	104	0.05	0.02 (0.89)	−0.34 (0.11)	1.47 (0.07)

(c) Low impulsivity (high consistency) and greater grey matter volume											
Middle frontal gyrus	R	BA8	32	23	43	3.83	1208	0.02	0.11 (0.39)	0.55 (0.005)	1.99 (0.02)
Middle frontal gyrus	L	BA46	−45	21	25	3.65	781	0.02	0.24 (0.07)	0.54 (0.007)	1.41 (0.08)
Middle frontal gyrus	R	BA6	22	10	53	3.40	62	0.03	0.13 (0.33)	0.56 (0.005)	1.97 (0.02)
Superior temporal gyrus	R	BA42	58	−12	13	4.38	2877	0.005	0.21 (0.11)	0.66 (0.001)	2.27 (0.01)
Middle temporal gyrus	R	BA39	56	−72	8	3.26	50	0.04	−0.17 (0.21)	0.37 (0.08)	2.19 (0.01)
Hippocampus	R	BA28	20	−21	−16	3.28	125	0.04	0.16 (0.22)	0.47 (0.02)	1.37 (0.09)
Post-central gyrus	L	BA2	−55	−19	24	4.52	1576	0.004	0.15 (0.24)	0.65 (0.001)	2.44 (0.007)
Posterior cingulate gyrus	L	BA23	−6	−31	29	4.45	365	0.005	−0.11 (0.39)	0.58 (0.003)	3.02 (0.001)
Posterior cingulate gyrus	R	BA31	9	−37	30	3.30	82	0.04	0.02 (0.88)	0.47 (0.02)	1.92 (0.03)
Occipital gyrus	R	BA11	9	48	−28	3.26	92	0.04	−0.04 (0.76)	0.57 (0.004)	2.7 (0.004)
Cuneus	L	BA17	−15	−73	10	3.26	94	0.04	−0.07 (0.59)	0.51 (0.01)	2.48 (0.007)

Significant voxels in patients											
(a) More attention to reward and greater grey matter volume											
Superior temporal gyrus	L	BA21	−50	9	−6	3.06	160	0.03	0.36 (0.004)	0.22 (0.3)	0.6 (0.27)
Thalamus	L		−5	−31	−4	4.00	1258	0.003	0.51 (<0.001)	−0.05 (0.82)	2.39 (0.008)

(b) Poor memory for past (relative to recent) outcomes and lower grey matter volume											
Superior frontal gyrus	R	BA8	20	35	40	3.73	146	0.006	−0.39 (0.002)	−0.19 (0.39)	0.86 (0.2)
Inferior temporal gyrus	R	BA20	55	−29	−17	3.18	208	0.02	−0.40 (0.002)	0.14 (0.52)	2.21 (0.01)

(c) Low impulsivity (high consistency) and greater grey matter volume											
Dorsolateral prefrontal cortex	R	BA9	46	21	26	3.60	1252	0.009	0.44 (0.001)	0.39 (0.06)	0.24 (0.41)
Superior temporal gyrus	L	BA22	−54	−31	12	3.47	1151	0.01	0.39 (0.002)	0.39 (0.06)	0.0 (1.0)
Posterior cingulate gyrus	R	BA31	14	−41	40	3.17	162	0.03	0.25 (0.05)	0.11 (0.6)	0.57 (0.28)

a MNI: Montreal Neurological Institute.
